# Relationship between physical function and sarcopenia in the older adults from Amazonas: A cross-sectional study

**DOI:** 10.1371/journal.pone.0320079

**Published:** 2025-03-19

**Authors:** Alex Barreto de Lima, Duarte Henrinques-Neto, David Scott, André de Araújo Pinto, Gustavo dos Santos Ribeiro, Miguel Peralta, Kessketlen Alves Miranda, Pedro Campos, Elvio Rúbio Gouveia

**Affiliations:** 1 Course of Physical Education, University of the State of Amazonas, Manaus, Amazonas, Brazil; 2 Skeletal Muscle Assessment Laboratory (LABSIM), Departament of Physycal Education, School of Tecnology and Sciences, São Paulo State University (UNESP), Presidente Prudente, Brazil; 3 Research Center in Sports Sciences, Health Sciences and Human Development, Maia University, Maia, Portugal; 4 Institute for Physical Activity and Nutrition (IPAN), School of Exercise and Nutrition Sciences, Deakin University, Burwood, Victoria, Australia; 5 School of Clinical Sciences at Monash Health, Monash University, Melbourne, Victoria, Australia; 6 State University of Roraima, Roraima, Brazil,; 7 Postgraduate Program in Rehabilitation Sciences, University Federal of Health Sciences of Porto Alegre, Porto Alegre, Brazil; 8 CIPER, Faculdade de Motricidade Humana, Universidade de Lisboa, Lisbon, Portugal; 9 Research Center in Physical Activity, Health and Leisure (CIAFEL), Faculty of Sport, University of Porto, Porto, Portugal; 10 Department of Informatics Engineering and Interactive Media Design, University of Madeira, Funchal, Portugal; 11 Department of Physical Education and Sport, University of Madeira, Funchal, Portugal; 12 LARSYS, Interactive Technologies Institute, Funchal, Portugal; University of Mississippi, UNITED STATES OF AMERICA

## Abstract

**Background:**

Physical functioning refers to the ability to perform daily living activities, namely basic activities, instrumental, and advanced activities. Poorer performance in these areas may indicate the potential presence of sarcopenia.

**Objectives:**

To analyze the differences in physical function between older people with and without sarcopenia and to investigate the associations between physical function tests and sarcopenia.

**Methods:**

A cross-sectional study based on data from older people from the Northern region of Brazil in the year 2018 was conducted. Study participants included 312 older people aged ≥  60 years (64.1% female). Sarcopenia was defined using the updated criteria of the European Working Group on Sarcopenia in Older People (EWGSOP2). Physical functioning was measured using functional physical fitness tests (30-second chair stand test, chair sit-and-reach test, 8-foot Up-and-Go Test, 6-minute walk test, 4-meter gait speed, and the Fullerton Advanced Balance Scale).

**Results:**

Confirmed sarcopenia was detected in 29.2% of participants, but no participant had severe sarcopenia. Most physical function parameters in the crude analysis were associated with confirmed sarcopenia (all p < 0.05), except for the back scratch test. In a model adjusted for sex, age and body mass index, slower 4-meter gait speed (OR = 1.29, 95%CI = 1.08 to 1.54), slower 8-foot up-and-go test time (OR = 1.32, 95%CI = 1.16 to 1.49), greater chair sit-and-reach test (OR = 0.97, 95%CI = 0.94 to 0.99) and higher self-reported Composite Physical Function scores (OR = 0.94, 95%CI = 0.89 to 0.99) were significantly associated with confirmed sarcopenia status.

**Conclusions:**

EWGSOP2 confirmed sarcopenia is prevalent in older people residing in Brazil’s Northern region and is independently associated with slower walking speed and chair rising ability, reduced trunk and lower-limb flexibility, as well as poorer self-reported physical function.

## Introduction

The aging process is accompanied by inherent physiological changes, which can lead to functional limitations that may reach the point where people cannot fully care for themselves [1]. These changes often manifest as alterations in body composition and muscle function in older people, in particular by decreasing muscle mass [2].

Starting as early as the third decade of life, the musculoskeletal system undergoes a gradual and continuous decline in muscle mass and strength [3]. The progressive decline in muscle mass, strength, and overall physical function can lead to physical disability and affect the performance of daily activities [4]. These physical and functional changes vary between sexes, with progression being slower in men and sharper in women, which is probably related to the coexistence of menopause [3]. This geriatric syndrome is worldwide known as sarcopenia [5]. It is estimated that about 10% of the population between 60-70 years has sarcopenia [6,7]. This prevalence can reach 50% in those aged 80 years or older [7,8], becoming a public health problem [9]. Due to the rapid aging worldwide, the burden of sarcopenia has increased significantly, which suggests the urgency of exploring effective strategies to prevent sarcopenia [10]. Early detection of older adults at risk for losing physical independence and a better understanding of the associated factors are pivotal for promoting healthy aging [1].

The European Working Group on Sarcopenia in Older People’s second consensus update (EWGSOP2) aimed to improve the definition, diagnosis, and sarcopenia treatment [11]. According to the EWGSOP2, low muscle strength is the main characteristic of sarcopenia (criterion I), which must have its diagnosis confirmed by low muscle mass (criterion II). Its severity can be identified by physical performance impairment (criteria III) [11]. In clinical practice it is possible to use low-cost tests like grip strength, muscle mass prediction, and gait speed for sarcopenia screening [12].

Although studies on sarcopenia have been conducted in cities in all Brazilian regions, there is little data available from the northern region of Brazil. To date, the available data comes mainly from some medium-sized cities in Amazonas and the sarcopenia status of small cities in the Amazon region remains unknown [13–16]. Furthermore, the northern Brazilian region stands out for being an area with different living conditions and difficult access to health services compared to other Brazilian regions [17]. It is characterized by dense forests, vast rivers, and a diverse cultural heritage including indigenous communities [18]. In addition to local customs that impact health conditions, with particular implications for the quality of life and lifestyle of the elderly [19]. The inhabitants of this region, especially those who live close to rivers, have access to less processed foods and tend to have a more active lifestyle, which can have protective effects on several aspects of their health, reflecting greater social vulnerability [20,21].

In this sense, the presence of sarcopenia and low physical function in the elderly population may represent challenge for assistance policies in the region. Although the literature reports a lack of agreement between different algorithms [3,11,12,22–27], to the best of our knowledge, no previous study has investigated differences in physical functioning between older people with and without EWGSOP2-defined sarcopenia in the northern region of Brazil. Thus, the purposes of this study were: (I) to analyze the differences in physical function between older people with and without sarcopenia and (II) to investigate the best physical function indicators to explain sarcopenia.

## Materials and methods

This was a cross-sectional study that included 312 elderly people (≥60 years of age) living in the community of Novo Aripuanã (Amazonas, Brazil). Participants were recruited in basic health units, parks, squares, churches, and other public places in the city’s urban area, in addition to invitations broadcast on local radio stations. Older adults living in rural areas were excluded from the study due to difficulties accessing the evaluation site (distance and transportation). After explanations about the procedures and risks of the study, all participants signed the informed consent form. All assessments were performed at Amazonas State University (UEA). The following criteria were considered for participant inclusion: (1) community-dwelling individuals aged 60 and above; (2) be independent in carrying out activities of daily living; (3) moderate or high level of cognitive function; (4) no contraindications for physical exertion (stroke, neurological diseases, unstable chronic conditions); (5) without chest pain, and/or angina pectoris and limiting joint pain [28]. The cognitive level was evaluated with the Mini-Mental State Examination (MMSE) [29]. An MMSE ≤ 15/30 points were used to exclude the study participants. This cross-sectional study was approved by the Ethics Committee of the UEA according to the Declaration of Helsinki [30] and Resolution 466/12 of the National Health Council, making part of the research project: “Sarcopenic Syndrome - Physical Function, Phenotype and Quality of life in elderly with and without sedentary lifestyle” (CAAE 74055517.9.0000.5016/ Referee 2.281.400). Sociodemographic data (age, marital status and education) were collected using a standardized questionnaire. Furthermore, the questionnaire from the Brazilian Association of Research Companies was applied to assess the socioeconomic level of the participants [31]. This instrument considers the possession of certain consumer goods, the head of the family, the presence of a domestic worker and access to public services.

Anthropometric indicators were carried out following the recommendations of the International Society for the Advancement of Kinanthropometry-ISAK [32]. Weight, height, body mass index (BMI), relaxed arm perimeter and skinfolds of the arm and medial thigh were evaluated. Weight was measured using a calibrated anthropometric scale, with participants barefoot and wearing light clothing. Height was measured using the metal stadiometer on the anthropometric scale. Participants were in an upright position, arms hanging at their sides and heels together. The BMI was calculated by the ratio between body mass and height squared (body mass/height^2^).

### Sarcopenia definitions

According to the EWGSOP2 definition, sarcopenia was diagnosed by combining the evaluation of muscle strength, muscle mass and physical performance [11]. In our study, no participant with severe sarcopenia was identified according to the EWGSOP2 criteria.

Muscle strength was measured using a manual digital dynamometer (EH101; Camry, Guangdong, China) [33]. Participants sat comfortably in a chair, with the forearm resting on the arm of the chair, flexed at 90 degrees and wrapping the hand being evaluated around the device handle [34]. Two measurements were taken alternately for each hand, using the highest value found among the four measurements. Values lower than 27 kg for men and 16 kg for women were adopted as the cutoff point to indicate “low muscle strength” [11]. Skeletal Muscle Mass (SMM) was estimated through Lee equation: Muscle Mass = Height(m) x (0.00744 x skinfold upperarm^2^ + 0.00088 x skinfold thigh^2^+ 0.00441 x calf girths^2^) + 2.4*sex - 0.048*age + race + 7.8, where R^2^= 0.91, P: < 0.0001, and SEE = 2.2 kg; sex = 0 for female and 1 for male, race = 0 for white and Hispanic. Values lower than 20 kg for men and 15kg for women were adopted as the cutoff point to indicate “low SMM” [35].

To evaluate physical performance, the 4-meter walking speed test (4-MGS) was applied [36]. The evaluator, using a stopwatch, recorded the time taken by the participants to cover a previously established distance of 4 meters. The test was performed twice, and the lowest value of the two measurements was considered and converted into walking speed. Values < 0.8 m/s were considered to indicate “low physical performance” [37]. The 4-MGS is one among several resources available to assess walking slowness and the severity of sarcopenia [38]. Considering the three aforementioned tests, participants were classified as “without sarcopenia and “with sarcopenia” (low strength and muscle mass). No participants with severe sarcopenia (low muscle strength, low SMM and low physical performance) were identified in our sample.

### Physical function

The Composite Physical Function (CPF) questionnaire, developed by Rikli & Jones [39], was used to assess the functionality of the elderly. The application was carried out through interviews with participants. It includes 12 activities, divided into 2 basic activities related to personal care, food and hygiene, 8 instrumental activities related to functioning in the community, such as household chores, shopping and getting around, and 2 advanced activities, such as domestic activities and practising activities/ more strenuous physical exercise. The score assigned corresponds to 0 (I cannot do it), 1 (I can do it with help) or 2 (I can do it on my own). The functionality was defined as high ( ≥ 24 points), moderate (14 to 23 points), or low ( < 14 points) [40].

Physical function was also assessed using physical fitness tests from the senior fitness test (SFT) battery [41]: (a) 30-second chair stand test (30-CST) (evaluate lower limb strength and endurance); (b) Chair sit-and-reach test (CSAR) (evaluate trunk and lower limbs flexibility); (c) Back Scratch Test (BST) (to evaluate upper limb flexibility); (d) 8-foot-up-and-go test (FUG) (evaluate agility and dynamic balance), and (e) Six-Minute Walk (6MWT) (to evaluate aerobic capacity).

Balance was assessed using the short version of the Fullerton Advanced Balance scale (FAB) [42]. The short version of the FAB Scale includes test items 4 (climbing and stepping over a 6-inch bench), 5 (tandem walking), 6 (one-sided stance), and 7 (standing on foam, eyes closed). of the original, which has a 10-item FAB scale. Each item is scored using a Likert scale from 0 (unable/unwilling to perform the test item) to 4 (correctly performs the test item). The total SF-FAB scale score ranges from 0 to 16, and participants who score 9 points or less are considered at high risk of falling [43].

### Statistical analysis

Data, including age, BMI, SMM, handgrip strength, CPF, 30-CST, CSAR, BST, FUG, 6MWT, 4-MGS, and FAB, were analyzed using descriptive statistics (mean, standard deviation, and 95% confidence intervals) for the overall sample and by sarcopenia groups. The normality was assessed using the Shapiro–Wilk test. The equality of variances was assessed using the Levene test. The t-test was interpreted according to the Levenne test. Before finishing, normality and the existence of outliers were assessed.

Differences between two groups of older adults (with and without sarcopenia) were evaluated using the student’s t-test. We conducted multivariable logistic regression analysis with physical function indicators and sarcopenia as the outcome. To assess the influence of different variables in the association between physical function and sarcopenia, we constructed four models: Model 1-adjusted for all indicators of physical function. Model 2- adjusted for sex, age, and BMI. Results from the regression analyses are presented as odds ratios (ORs) with 95% confidence intervals (CIs). The variance inflation factor for each independent variable was calculated to assess multicollinearity. To identify potential multicollinearity, a cut-off point of ≥ 5.0 was considered. The IBM SPSS Statistics version 28 (IBM Corp., Armonk, NY) was used for statistical analyses considering p < 0.05 was statistically significant.

## Results

The study population consisted of 312 older adults living in the community of Northern Brazil (64.1% women) with a mean age of 72.4 ±  8.1 years. It was observed that 70.2% of the sample (68.8% men; 71% women) had probable sarcopenia (low muscle strength) and 22.8% had low gait speed (15.2% men; 27% women). Overall, 29.2% of participants had confirmed sarcopenia according to EWGSOP2 criteria. In summary, the sarcopenic group was older and had higher BMI, and worse CPF and physical function than counterparts without sarcopenia. Table 1 presents the sample characteristics.

**Table 1:** Descriptive characteristics of the participants.

Variable	Overall (n = 312)		Sarcopenic (n = 91)		Non-sarcopenic (n = 221)		*Mean difference (IC 95%)*	*p*-value
	Mean ± SD	CI	Mean ± SD	CI	Mean ± SD	CI		
Age, years	72.6 ± 7.8	71.8 to 73.5	76.3 ± 8.6	74.5 to 78.0	71.1 ± 6.9	70.2 to 72.1	5.2 (3.1 to 7.3)	0.001
Height (m)	1.53 ± 0.1	1.53 to 1.55	1.52 ± 0.1	1.50 to 1.53	1.54 ± 0.1	1.53 to 1.55	-0.02 (-0.03 to -0.01)	0.022
Weight (Kg)	63.7 ± 0.7	62.3 to 65.1	59.6 ± 1.3	57.1 to 62.2	65.4 ± 0.8	63.7 to 67.0	-5.8 (-8.1 to -3.5)	<0.001
BMI, kg/m^2^	26.9 ± 4.7	26.4 to 27.4	25.6 ± 4.6	24.6 to 26.6	27.4 ± 4.6	26.8 to 28.0	-1.8 (-3.0 to -0.7)	0.002
**Circumferences**								
Upper arm (cm)	28.3 ± 0.2	27.8 to 28.7	26.7 ± 0.4	25.9 to 27.5	28.9 ± 0.3	28.3 to 29.5	-2.2 (-3.0 to -1.4)	<0.001
Thigh (cm)	46.4 ± 0.3	45.8 to 47.0	44.5 ± 0.5	43.5 to 45.5	47.2 ± 0.4	46.4 to 48.0	-2.7 (-3.9 to -1.5)	<0.001
Calf (cm)	32.8 ± 0.2	32.4 to 33.2	31.8 ± 0.4	31.1 to 32.5	33.2 ± 0.2	32.8 to 33.6	-1.4 (-2.2 to -0.6)	<0.001
Skeletal Muscle mass, kg	19.9 ± 4.6	19.3 to 20.4	18.4 ± 4.0	17.6 to 19.3	20.4 ± 4.4	19.8 to 21.1	-2.0 (-3.2 to -0.8)	0.001
**Physical Function**								
Handgrip strength, kg	23.7 ± 9.2	22.6 to 24.7	16.4 ± 5.7	15.2 to 17.6	26.7 ± 8.6	25.6 to 27.9	-10.3 (-12.7 to -7.9)	0.001
CPF, score	19.5 ± 4.8	18.9 to 19.9	18.1 ± 5.4	16.9 to 19.2	20.0 ± 4.4	19.4 to 20.6	-1.9 (-3.3 to -0.5)	0.001
30-CST, n	10.9 ± 3.2	10.5 to 11.2	10.1 ± 2.9	9.5 to 10.7	11.2 ± 3.3	10.1 to 11.6	-1.1 (-1.9 to -0.3)	0.006
CSAR, cm	4.7 ± 11.4	3.5 to 6.0	1.2 ± 12.7	-1.4 to 3.9	6.2 ± 10.6	4.8 to 7.6	-5.0 (-8.3 to -1.7)	0.001
BST, cm	-16.9 ± 14.7	-18.6 to -15.3	-19.5 ± 14.9	-22.6 to -16.3	-15.9 ± 14.6	-17.9 to -14.0	-3.6 (-7.4 to 0.2)	0.057
FUG, sec	8.1 ± 2.7	7.8 to 8.4	9.6 ± 3.4	8.9 to 10.3	7.5 ± 1.7	7.2 to 7.7	2.1 (1.3 - 2.9)	0.001
6MWT, m	407.3 ± 108.4	395.2 to 419.4	372.8 ± 94.3	353.2 to 392.4	421.5 ± 110.8	406.8 to 436.2	-48.7 (-71.9 to -25.5)	0.001
4-MGS, m/s.	1.09 ± 0.36	1.05 to 1.13	0.93 ± 0.31	0.87 to 0.99	1.15 ± 0.36	1.12 to 1.20	-0.22 (-0.30 to -0.14)	<0.001
FAB, score	12. 4 ± 3.7	12.0 to 13.0	11.5 ± 4.3	10.6 to 12.4	12.8 ± 3.3	12.4 to 13.3	-1.3 (-2.2 to -0.4)	0.003

**Legend:** SD, standard deviation; CI, confidence interval; BMI, body mass index; CPF, Composite Physical Function; 30-CST, 30-second chair stand test; CSAR, chair sit-and-reach test; BST, back scratch test; FUG, 8-foot up-and go test; 6MWT, 6-minute walk test; 4-MGS, 4-meter gait speed; FAB, Fullerton Advanced Balance. Values expressed as Mean ±  SD and 95%CI.

To adjust for confounding covariates that affect sarcopenia, we applied two multi-variable logistic regression models. Table 2 presents the association between sarcopenia and the physical function in regression models. Higher values of CPF, CST, CSAR, 6MWT, and FAB were associated with lower likelihood for sarcopenia in an unadjusted model 1 (OR 0.89 to 0.99; p < 0.05). In this model, higher values of FUG and 4-MGS were associated with higher likelihood of sarcopenia. In the adjusted model, it was observed that higher values of FUG (OR 1.32 to 1.41, p < 0.001) and 4-MGS (OR 1.29 to 1.47; p < 0.05) were associated with higher likelihood of having sarcopenia.

**Table 2:** Association between sarcopenia and physical function unadjusted to confounding covariates.

Physical function	Unadjusted model				Adjusted model			
	β	*p*	OR	95%CI	β	*p*	OR	95%CI
CPF, score	-0.083 *	0.001	0.92	0.88 to 0.97	-0.061 *	0.040	0.94	0.89 to 0.99
30-CST, n	-0.114 *	0.007	0.89	0.82 to 0.97	-0.075	0.085	0.93	0.85 to 1.01
CSAR, cm	- 0.039 *	0.001	0.96	0.94 to 0.98	-0.034 *	0.008	0.97	0.94 to 0.99
BST, cm	-0.017	0.058	0.98	0.97 to 1.00	-0.015	0.125	0.99	0.97 to 1.00
FUG, sec	0.320 *	0.001	1.38	1.23 to 1.54	0.276 *	0.001	1.32	1.16 to 1.49
6MWT, m	-0.005 *	0.001	0.99	0.99 to 0.99	-0.004 *	0.003	0.99	0.99 to 0.99
4-MGS, m/s	0.357 *	0.001	1.43	1.21 to 1.69	0.254 *	0.006	1.29	1.08 to 1.54
FAB, score	-0.098 *	0.003	0.90	0.85 to 0.97	-0.061	0.118	0.94	0.87 to 1.02

**Legend** Unadjusted model; Adjusted model, adjusted model for sex, age, and body mass index; OR, odds ratio; CI, Confidence interval; CPF, Composite Physical Function; CST, 30-second chair stand test. CSAR, chair sit-and-reach test. BST, back scratch test. FUG, foot up-and-go test. 6MWT, 6-minute walk test. 4-MGS, m/s, 4-meters gait speed; FAB, Fullerton Advanced Balance Scale. *  p-value below 0.05.

## Discussion

This study demonstrated that sarcopenia is prevalent among older adults residing in the Amazonas region of northern Brazil, with almost 30% meeting EWGSOP2 criteria for confirmed sarcopenia. Furthermore, older adults with sarcopenia demonstrated a number of physical function limitations, including slower walking speed and chair rising ability, reduced trunk and lower-limb flexibility, as well as poorer self-reported physical function, that likely impact their capacity for independent living.

Sarcopenia is one of the symptoms of the biological process of ageing, which is characterised by decreased SMM. The reduction of SMM leads to a decrease in functional capacity, as well as an increase in the incidence of pathologies, particularly non-communicable pathologies [1,44,45]. Our findings show that the elderly population studied had a high prevalence of sarcopenia (about 30% of the sample) when compared with international populations [46], as well as in Brazilian populations as from in the interior of São Paulo (17.5%) [47], Nova Santa Rita (23.7%) [48], Macapá (6.1%) [49], Natal (4.6%) [50], or Manaus [51]. Notwithstanding, It is crucial to recognise that variations in the prevalence of sarcopenia can arise due to differences in diagnostic criteria and cut-off points for SMM in populations with specific characteristics, such as age, geographical region, individual perception of health, the presence or not of communicable diseases, malnutrition and socioeconomic status [52]. This emphasises the need to adapt the diagnostic criteria to the specific ethnic groups under study [53]. It should also be noted that, according to the EWGSOP2 criteria, no cases of severe sarcopenia were identified, which suggests that the criteria may not be specific and sensitive for this specific population.

Scientific studies have shown that assessing functional fitness can indicate the risk of sarcopenia [54,55]. Our findings highlight the predominant functional determinants elucidating sarcopenia among the elderly residing in Northern Brazil, focusing on variables associated with mobility proficiency over brief periods. Specifically, the CPF, CSAR, 4-MGS and FUG tests emerged as key indicators, independent of sex, age, and BMI. When considering a training programme for treating sarcopenia, it should be considered that there are different types of exercise, and a single or mixed programme can be planned combining the different types of exercise [3]. Although some consensus and research for the treatment of sarcopenia emphasize multicomponent [56], strength [57], and power 58] physical exercise programmes, evidence to support the inclusion of power training in exercise programmes for older people with sarcopenia is limited [59].

These findings can have important implications for the early detection and effective management of sarcopenia in the elderly in northern Brazil and can direct future interventions and preventive strategies in public health policies.

## Strengths and limitations

This study constitutes a robust contribution to scientific investigation, particularly within the domain of public health concerning populations characterized by specific geographical, cultural, and social attributes. Notably, the adoption of the contemporary criteria outlined by the EWGSOP2 for the delineation of sarcopenia. By aligning with current guidelines, this approach solidifies the diagnostic foundation and enhances the credibility of the outcomes. Secondly, the geographical, social and biological characteristics of this sample are very specific, and there aren’t many scientific studies with samples with these characteristics.

On the other hand, this study is not without its limitations. Firstly, the research design is cross-sectional, which precludes drawing causal inferences. Another limitation is the representativeness of the sample. Since the sample is not representative of all the regions in the interior of the state of Amazonas, it is not possible to generalize the results. These limitations warrant consideration when interpreting and applying the findings to broader contexts.

## Conclusions

Our results demonstrate that EWGSOP2 confirmed sarcopenia is common in older adults living in the Northern of Brazil, and that those with sarcopenia have greater likelihood of poor performance in a range of functional assessments. These assessments are relevant in public health monitoring due to their ease of application and low cost, and may support efforts to prevent falls and sarcopenia in populations with lower economic resources.

## Supporting information

S1 File (XLSX)
