# Correction: Relationship between physical function and sarcopenia in the older adults from Amazonas: A cross-sectional study

**DOI:** 10.1371/journal.pone.0327300

**Published:** 2025-06-27

**Authors:** Alex Barreto de Lima, Duarte Henrinques-Neto, David Scott, André de Araújo Pinto, Gustavo dos Santos Ribeiro, Miguel Peralta, Kessketlen Alves Miranda, Pedro Campos, Elvio Rúbio Gouveia

There are errors in the author affiliations. The correct affiliations are as follows:

Alex Barreto de Lima^1,2^, Duarte Henrinques-Neto^3^, David Scott^4,5^, André de Araújo Pinto^6^, Gustavo dos Santos Ribeiro^7^, Miguel Peralta^8^, Kessketlen Alves Miranda^9^, Pedro Campos^10,11,12,13^, Elvio Rúbio Gouveia^11,13^

**1** Course of Physical Education, University of the State of Amazonas, Manaus, Amazonas, Brazil, **2** Skeletal Muscle Assessment Laboratory (LABSIM), Departament of Physycal Education, School of Tecnology and Sciences, São Paulo State University (UNESP), Presidente Prudente, Brazil, **3** Research Center in Sports Sciences, Health Sciences and Human Development, Maia University, Maia, Portugal, **4** Institute for Physical Activity and Nutrition (IPAN), School of Exercise and Nutrition Sciences, Deakin University, Burwood, Victoria, Australia, **5** School of Clinical Sciences at Monash Health, Monash University, Melbourne, Victoria, Australia, **6** State University of Roraima, Roraima, Brazil, **7** Postgraduate Program in Rehabilitation Sciences, University Federal of Health Sciences of Porto Alegre, Porto Alegre, Brazil, **8** CIPER, Faculdade de Motricidade Humana, Universidade de Lisboa, Lisbon, Portugal, **9** Research Center in Physical Activity, Health and Leisure (CIAFEL), Faculty of Sport, University of Porto, Porto, Portugal, **10** Department of Informatics Engineering and Interactive Media Design, University of Madeira, Funchal, Portugal, **11** LARSYS, Interactive Technologies Institute, Funchal, Portugal, **12** WoWSystems Informática Lda, Funchal 9050-100, Portugal, **13** Department of Physical Education and Sport, University of Madeira, Funchal, Portugal.

There are errors in the Funding statement. The correct Funding statement is as follows: This study was funded by the Portuguese Recovery and Resilience Programme (PRR), IAPMEI/ANI/FCT, under the Agenda C645022399-00000057 (eGamesLab). E.R.G. and P.C. are supported by the following FCT projects: 10.54499/LA/P/0083/2020, 10.54499/UIDP/50009/2020, and 10.54499/UIDB/50009/2020.
